# ResGRU: A Novel Hybrid Deep Learning Model for Compound Fault Diagnosis in Photovoltaic Arrays Considering Dust Impact

**DOI:** 10.3390/s25041035

**Published:** 2025-02-09

**Authors:** Xi Liu, Hui Hwang Goh, Haonan Xie, Tingting He, Weng Kean Yew, Dongdong Zhang, Wei Dai, Tonni Agustiono Kurniawan

**Affiliations:** 1 School of Electrical Engineering, Guangxi University, Nanning 530004, China; lx2212392074@st.gxu.edu.cn (X.L.);; 2 School of Engineering, Taylor’s University Lakeside Campus, Subang Jaya 47500, Malaysia; 3 Department of Electrical Engineering, School of Engineering & Physical Sciences, Heriot-Watt University Malaysia Campus, Putrajaya 62200, Malaysia; 4 College of the Environment and Ecology, Xiamen University, Xiamen 361102, China; tonni@xmu.edu.cn

**Keywords:** photovoltaic (PV) system, fault diagnosis, dust impact, I-V curve, residual network (ResNet), bidirectional gated recurrent unit (BiGRU)

## Abstract

With the widespread deployment of photovoltaic (PV) power stations, timely identification and rectification of module defects are crucial for extending service life and preserving efficiency. PV arrays, subjected to severe outside circumstances, are prone to defects exacerbated by dust accumulation, potentially leading to complex compound faults. The resemblance between individual and compound faults sometimes leads to misclassification. To address this challenge, this paper presents a novel hybrid deep learning model, ResGRU, which integrates a residual network (ResNet) with bidirectional gated recurrent units (BiGRU) to improve fault diagnostic accuracy. Additionally, a Squeeze-and-Excitation (SE) module is incorporated to enhance relevant features while suppressing irrelevant ones, hence improving performance. To further optimize inter-class separability and intra-class compactness, a center loss function is employed as an auxiliary loss to enhance the model’s discriminative capacity. This proposed method facilitates the automated extraction of fault features from I-V curves and accurate diagnosis of individual faults, partial shading scenarios, and compound faults under varying levels of dust accumulation, hence aiding in the formulation of efficient cleaning schedules. Experimental findings indicate that the suggested model achieves 99.94% accuracy on pristine data and 98.21% accuracy on noisy data, markedly surpassing established techniques such as artificial neural networks (ANN), ResNet, random forests (RF), multi-scale SE-ResNet, and other ResNet-based approaches. Thus, the model offers a reliable solution for accurate PV array fault diagnosis.

## 1. Introduction

Given the intensification of fossil fuel depletion and environmental degradation, there is an urgent necessity for sustainable and environmentally friendly energy solutions to address the escalating energy demands and conservation challenges [1]. Solar energy has attracted considerable interest among renewable energy sources due to its sustainability, absence of pollution, and extensive availability. Photovoltaic (PV) power generation has emerged as the primary means of utilizing solar energy resources. Despite the COVID-19 pandemic, global PV electricity generation rose by 18% in 2021, attaining 1000 TWh [2]. As of 2024, the global installed capacity of solar power plants has surpassed 1400 GW [3].

PV arrays, consisting of numerous PV modules and intricate wiring, are essential components of PV systems. They are generally deployed in severe outside conditions and are vulnerable to diverse types and degrees of defects caused by numerous potential variables, such as high winds, precipitation, hail, dust, ultraviolet radiation, thermal cycling, transportation incidents, and others [4]. These defects may include open-circuit faults (OCF), abnormal degradation (AD), partial shading (PS), line-line faults (LLF), micro-cracks, and others. To safeguard PV systems from severe damage, conventional protective devices, such as ground fault protection devices (GFPDs) and overcurrent protection devices (OCPDs), are commonly installed on the direct current side of the system [5]. However, the nonlinear behavior of PV arrays, high fault impedance, low mismatch faults, and low irradiance levels can result in the failure of protective devices [6]. Moreover, dusty surroundings substantially affect the PV systems’ operational efficiency [7,8]. Importantly, prolonged dust deposition can complicate signal faults, leading to compound faults that make fault diagnosis more challenging. Consequently, efficient fault detection and diagnosis (FDD) of PV systems in dusty conditions is crucial to maintaining the reliability and safety of PV arrays.

In recent years, various fault diagnostic approaches have been introduced to enhance PV systems’ FDD. These methodologies can be classified as model-driven and data-driven approaches. Model-driven approaches involve modeling the PV system and comparing it with the actual monitored outputs to assess fault status. The modeling can be classified into five categories: empirical formulas [9], equivalent circuits [10], machine learning [11], fuzzy inference systems [12], and hybrid techniques [13]. The accuracy of the PV system modeling directly influences the precision of fault diagnosis. When multiple faults occur, due to the nonlinear coupling among these faults, their interaction mechanisms are challenging to comprehend. Therefore, modeling becomes more complex, necessitating the utilization of advanced diagnostic algorithms for the extraction of deep feature insights. The advent of new digital infrastructures has contributed to the increasing use of data-driven approaches in FDD for PV systems. Data-driven approaches can be categorized into three categories based on the type of data: image-based methods, measured string current or voltage-based methods, and measured current–voltage (I-V) curve-based methods [14].

Image-based methods employ machine learning and image analysis techniques to assess diverse images, including electroluminescence (EL) images, infrared images, and visible light, for the identification of flaws in PV modules, such as oxygen, hotspots, and PS [15]. Juan and Kim et al. utilized support vector machine (SVM) classification to design a binary classifier capable of distinguishing between defective and normal solar cells from EL images [16]. Schuss et al. employed synchronized thermal and time-resolved thermal imaging techniques to localize regions of interest in infrared image datasets for the FDD of PV arrays [17]. Rico Espinosa et al. developed a convolutional neural network (CNN)-based method for automatically identifying physical faults in PV stations. The approach segments and semantically classifies RGB images, achieving an accuracy of up to 75% for two output classes [18]. However, these images often require collection via high-resolution cameras and unmanned aerial vehicles (UAVs), resulting in considerable expenses.

Methods based on string current or voltage typically rely on measurements obtained from sensors positioned on the direct current side of PV arrays. In [19], Chen Z et al. utilized string current and array voltage as input features, integrating them with the random forest (RF) ensemble learning algorithm for the early identification of PV faults. The study focused solely on individual faults, which were categorized into four types: LLF, PS, AD, and OCF. In [20], Van Gompel J et al. utilized PV array voltage and current alongside environmental factors as input features to develop a model based on gated recurrent units (GRU). This model is capable of accurately classifying six common individual faults and previously unseen faults in PV systems. In [21], Momeni H et al. employed string current and voltage as input features and introduced an enhanced graph-based semisupervised learning algorithm to classify and locate LLF and OCF. However, the measurement of current and voltage is entirely reliant on sensors, resulting in an increase in sensor quantity and, subsequently, operating and maintenance costs. Moreover, different types of faults may lead to similar changes in current and voltage, thereby affecting the accuracy of FDD.

The method based on I-V curves of PV arrays is the primary approach in this study, which offers comprehensive information for detecting fault states. Recent advancements in I-V curve scanning capabilities in PV inverters have enabled real-time defect diagnosis without the need for supplementary hardware. Consequently, researchers have increasingly focused on extracting local features from I-V curves as inputs for FDD models. For instance, in [22], Fadhel et al. employed *I_mpp_*, *V_mpp_*, and *P_mpp_* to distinguish between four different shading scenarios. In [23], Chen Z et al. extracted key features, including *I_mpp_*, *V_mpp_*, *V_oc_*, and *I_sc_*, and applied them as inputs to a kernel extreme learning machine (KELM) for the identification of AD, LLF, OCF, and PS. Additionally, similar approaches have been proposed in [24,25]. However, it is important to acknowledge that this research employed only partial curve data, which limits their ability to diagnose complex faults such as those caused by dust pollution. Furthermore, these methods rely heavily on manual feature extraction and expert knowledge, which lack automation and may overlook valuable features, thus reducing accuracy. Therefore, some machine learning models have also employed the entire I-V curve as input. For example, [26] fully leveraged raw I-V curves to achieve 100% diagnostic accuracy for PS, LLF, OCF, and AD. In [27], Chen Z et al. combined I-V curves with environmental factors as inputs to a deep residual network (ResNet) for the automatic identification of AD, LLF, OCF, and PS. Although using the entire I-V curve can enhance diagnostic accuracy, it requires more precise diagnostic models.

In summary, the aforementioned PV fault diagnosis approaches exhibit several advantages and limitations, as summarized in Table 1. Compared with other methods, the use of entire I-V curves is more effective for diagnosing multiple faults and achieving a higher fault detection rate. Furthermore, these methods primarily focus on conventional individual faults while often neglecting more complex and realistic compound faults caused by dust accumulation in operational environments. These faults are common, and due to the resemblance in electrical characteristics, they are challenging to distinguish accurately. Moreover, these approaches fail to consider the varying levels of dust accumulation on PV arrays, which can enhance the formulation of efficient cleaning schedules. To mitigate this challenge, an effective feature extraction method is needed, as the fault features caused by dust accumulation are intricate. Consequently, this paper proposes a novel diagnostic model that integrates the ResNet architecture with a bidirectional gated recurrent unit (BiGRU), incorporating a Squeeze-and-Excitation (SE) module along with a center loss function for optimization. This model directly takes the I-V curve waveform as input, eliminating the need for manual feature extraction, and demonstrates high accuracy in identifying individual and compound faults in PV arrays.

Overall, the most important contributions of this work are as follows:

- Various fault tests were executed in Simulink to capture I-V curves under diverse fault scenarios. This research, in contrast to prior studies, incorporates an investigation of compound faults under varying levels of dust accumulation. The feasibility of using I-V curves for fault diagnosis was demonstrated, and bilinear interpolation was applied to downsample the curves to reduce data redundancy.
- The proposed model integrates ResNet with BiGRU and the SE block to extract information from both spatial and temporal dimensions, enhancing diagnostic accuracy. The SE module enhances feature representation by amplifying informative features and suppressing irrelevant ones. Furthermore, we employ center loss as an auxiliary loss function to extract deep features with intra-class compactness and inter-class separation.
- Given the irreversible damaging characteristics of fault tests, noise was introduced to simulate real-world conditions. Both simulation and noise experiments confirmed the model’s exceptional accuracy, reliability, and generalization capabilities. Comparative analyses with other models further validated its superior diagnostic performance.

The subsequent sections of this work are structured as follows: Section 2 illustrates the PV model and its related faults, along with an analysis of the effects of various faults on the I-V characteristic curves. Section 3 presents the proposed model architecture and diagnostic method. Section 4 provides the diagnostic results, along with a comparison with existing methods in the literature. Finally, Section 5 closes the analysis and addresses prospective research directions.

## 2. Simulation and Fault Characteristic Analysis of PV System

### 2.1. The Configuration of Grid-Connected PV System

**Figure 1:**
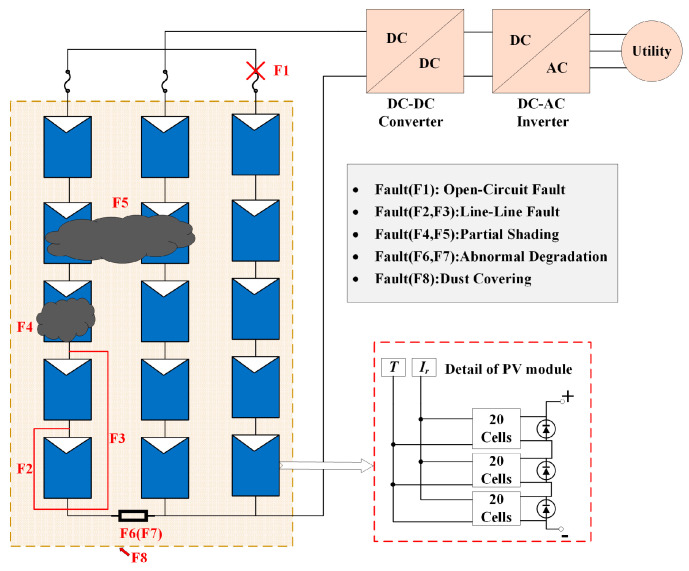
Configuration diagram of grid-connected PV system with various faults.

A typical grid-connected PV system, generally including a PV array, an inverter, and a direct current junction box, is depicted in Figure 1. The configurations of the PV array can be implemented using methods including series, honeycomb, bridge-linked, total-cross-tied, and series-parallel, with the series-parallel method being the most commonly employed [28]. To optimize the system’s power generation efficiency, the inverter usually incorporates a maximum power point tracking module [29]. The internal configuration of the PV module is illustrated by the red dashed line in Figure 1. Each module comprises 60 cells arranged in series, with bypass diodes linked in parallel across the cells to avoid damage from the hot-spot effect.

### 2.2. The PV Simulation Model for Fault Analysis

**Figure 2:**
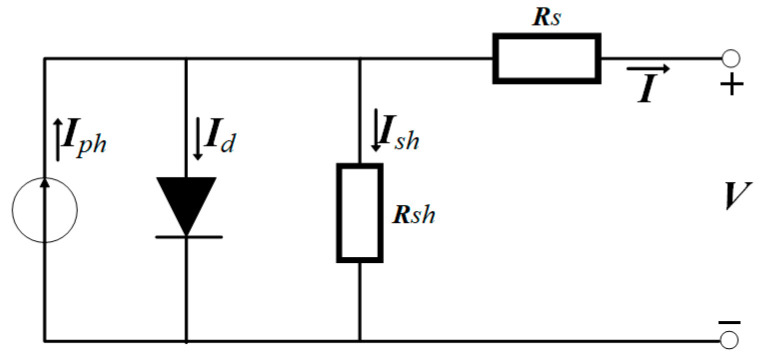
The single diode model.

Until now, many modeling methods for PV modules with different levels of complexity and accuracy have been proposed. The single diode model is widely adopted for its simplicity and accuracy [30]. The single diode model of PV cells is shown in Figure 2, and the I-V equation can be expressed as follows:

(1) $$I=I_{ph}-I_{s}[\exp(\frac{q(V+R_{s}I)}{nkT})-1]-\frac{V+R_{s}I}{R_{sh}},$$

where *V* and *I* are the output voltage and current of the PV cell, *I_ph_* is the photogenerated current, *q* is the electron charge, *I_s_* is the saturation current of the diode, *n* is the diode ideality factor, *k* is the Boltzmann constant, *T* is the cell temperature, and *R_s_* and *R_sh_* are the series and shunt resistance of the module, respectively.

**Figure 3:**
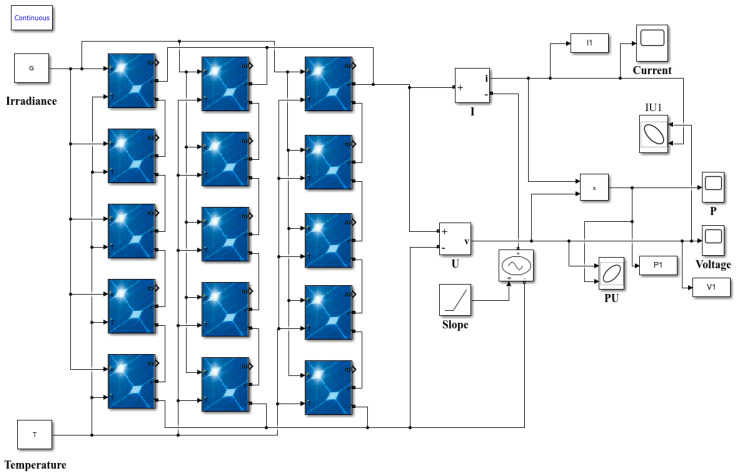
PV array simulation model.

Hence, a PV array model has been developed under MATLAB Simulink (R2022b) based on the single diode model, as shown in Figure 3. The array is composed of 15 ASMS-220P modules, configured in three parallel strings, with each string containing five modules connected in series. Table 2 and Table 3 provide the detailed parameters for the modules and the array, respectively. *I_SC_* represents the short-circuit current, and *V_OC_* refers to the open circuit voltage. *I_MPP_* and *V_MPP_* refer to the current and voltage at the maximum power point, respectively. *β*_rel_ and *α*_rel_ are the temperature coefficient of *V_OC_* and the temperature coefficient of *I_SC_*, respectively.

### 2.3. Fault Characteristic Analysis of PV System

In this study, four common fault types are investigated, namely OCF, LLF, PS, and AD, which are commonly encountered by PV arrays, particularly in complex outdoor environments [31]. Additionally, the performance of PV systems is significantly influenced by dusty conditions. Since dust coverage (DC) is an unavoidable issue for PV arrays, the faults influenced by this condition are considered compound faults of PV arrays, which are frequently encountered in practical applications.

**Figure 4:**
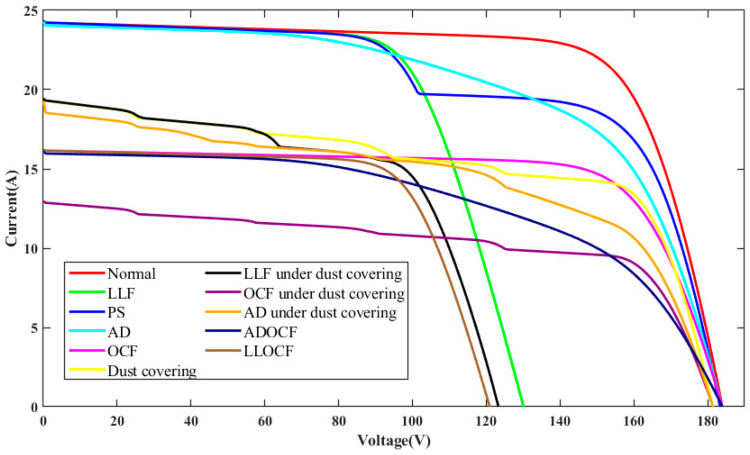
I-V characteristics curves of different faults under STC.

The I-V characteristic curves for PV individual faults and compound faults under standard test condition (STC) are presented in Figure 4. It can be seen that the individual faults studied in the PV array include OCF, PS, LLF, AD, and DC. OCF primarily occurs on fractured PV cells and modules or inside the interconnections of modules. When a string in the PV array experiences OCF, the short-circuit current will be reduced, while the open-circuit voltage of the PV array will remain mostly unchanged. The PS fault denotes the irregular distribution of input irradiance on the PV array plane, resulting from factors such as buildings, dust, birds, etc. Compared to normal conditions, the I-V curve under PS fault exhibits many peaks and inflection points, which result from the bypass diode. LLF in a PV string is usually caused by unintended connections between two points of different potentials, leading to a significant drop in both the open-circuit voltage and maximum power point voltage. However, the short-circuit current basically remains unchanged. The fault of AD can result from various circumstances, including mechanical damage, cable deterioration, and harsh operating conditions, which lead to corrosion and discoloration of PV modules. Compared to normal conditions, the closed area of the I-V curve is reduced. For PV arrays under DC, it is considered to be a particular type of shading that impacts all modules within the array [32]. However, the intensity of DC varies across each module. Consequently, due to the uneven distribution of dust, each module experiences varying levels of shading, causing the array’s characteristic curve to exhibit a different number of peaks. The current amplitude diminishes more rapidly than under normal conditions, and the short-circuit current amplitude is also reduced.

The compound faults studied in the PV array include OCF, LLF, and AD under various levels of DC. In the presence of DC, PS faults usually show the characteristics of uneven dust, which are not taken into account. According to the above dust characteristics, these features will also be superimposed on these individual faults (OCF, LLF, AD) to form compound fault features. In addition, to expand the scope beyond solely studying compound faults under dust coverage, this paper also considers two compound faults without dust coverage: the concurrent occurrence of LLF and OCF (referred to as LLOCF) and the concurrent occurrence of AD and OCF (referred to as ADOCF). Compared to normal conditions, the I-V curves for LLOCF and ADOCF in Figure 4 demonstrate a considerable reduction in open-circuit voltage and short-circuit current. Therefore, the I-V curve may serve as the input for a compound FDD model.

**Figure 5:**
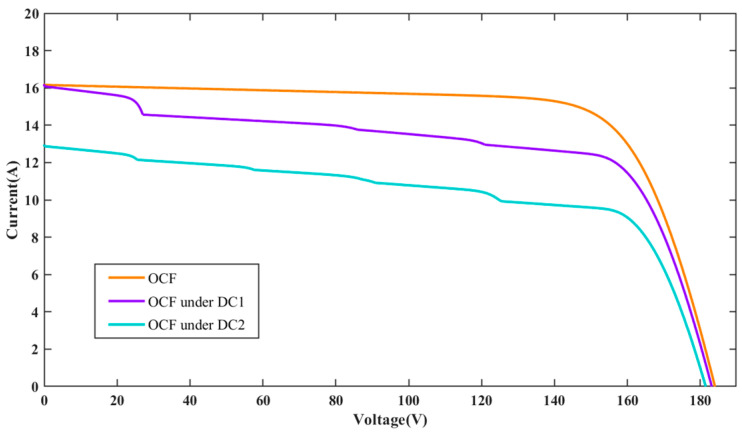
I-V characteristic curves for OCF under different dust coverage levels.

**Figure 6:**
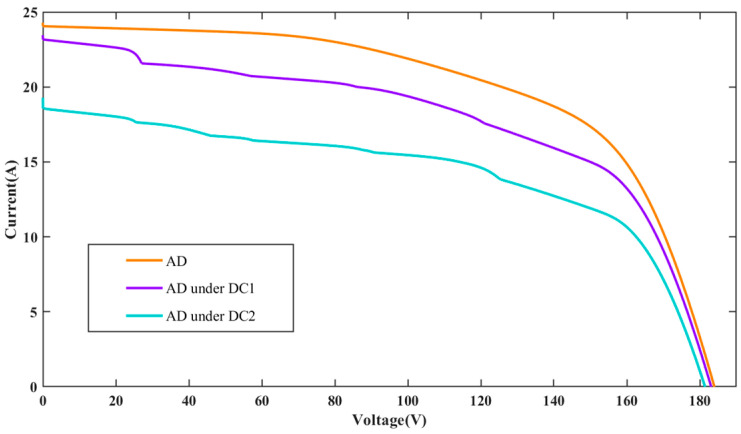
I-V characteristic curves for AD under different dust coverage levels.

**Figure 7:**
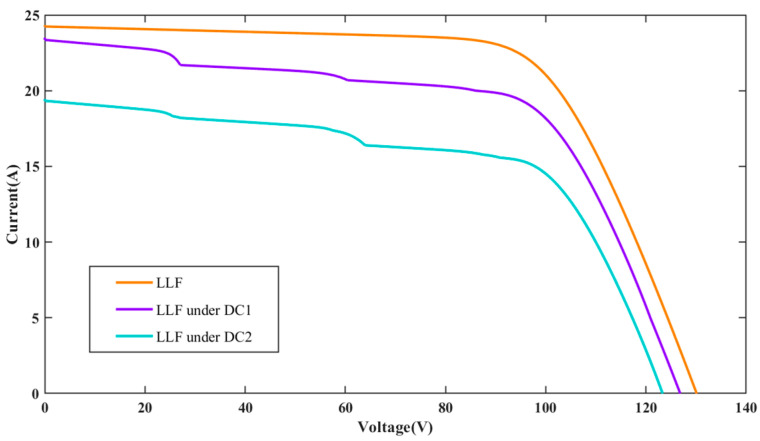
I-V characteristic curves for LLF under different dust coverage levels.

To further evaluate the impact of different DC levels on the I-V characteristics, two different DC levels are investigated in this paper. These levels are modeled by randomly adjusting the irradiance gain amplifiers of all PV panels to [0.8, 1] and [0.6, 0.8] in the simulation model, referred to as dust coverage 1 (DC1) and dust coverage 2 (DC2), respectively. Figure 5, Figure 6 and Figure 7 present the I-V curves for OCF, AD, and LLF under these two DC levels. It is observed that the I-V curves for the same type of compound faults show similar trends under different DC levels, making classification more difficult. Fortunately, the area encompassed by the I-V curve can be different. The total I-V curve under DC2 is lower compared to DC1. The above results highlight that different fault conditions significantly influence the I-V characteristic curves, underscoring the potential viability of the FDD model based on these curves.

## 3. Methodology

### 3.1. Data Preprocessing

To prepare high-quality data for training the FDD model, the raw I-V curves and associated environmental conditions undergo a preprocessing process consisting of two stages. The first stage involves downsampling the raw I-V curves to reduce data redundancy. The second stage is to normalize the sample features before using them as input to the proposed model.

**Figure 8:**
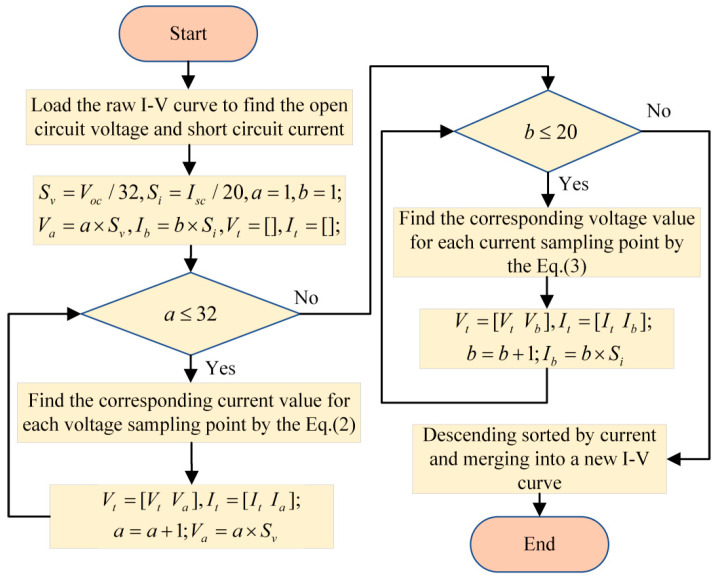
Flowchart of the resampling method.

Most of the raw I-V curves collected from I-V testers contain a large quantity of data points. To minimize data redundancy, a technique involving linear resampling of the raw I-V curves was employed, utilizing a current and voltage grid with equal spacing. As a result, the total number of data points in each I-V curve was eventually diminished from approximately 200 to 52. The steps involved in this resampling method are depicted in Figure 8 and described as follows.

Initially, ascertain the short-circuit current *I_SC_* and the open-circuit voltage *V_OC_*. Since the curve sampling points are discrete and there may be no real open-circuit and short-circuit points, *V_OC_* is determined from the peak voltage point in the curve data, and *I_SC_* is determined from the peak current point in the curve data. Then, 32 new data points are resampled in the range [0, *V_OC_*] according to the voltage equal interval, where *V_a_* = *a*$\frac{V_{OC}}{32}$ (*a* = 1, 2, ⋯, 32) represents the resampled voltage value of the *a*th point. Finally, the current value *I_a_* corresponding to *V_a_* is obtained by bilinear interpolation, as shown in Equation (2). Similarly, 20 new data points are resampled in the range of [0, *I_SC_*] according to the current equal interval, where *I_b_* = *b*$\frac{I_{SC}}{20}$ (*b* = 1, 2, ⋯, 20) represents the resampled current value of the *b*th point. Finally, bilinear interpolation is then used to obtain the corresponding voltage value *V_b_*, as shown in Equation (3).

(2) $$I_{a}=\frac{(V_{a}-V_{1})\times I_{2}+(V_{2}-V_{a})\times I_{1}}{V_{2}-V_{1}},$$

(3) $$V_{b}=\frac{(I_{b}-I_{1})\times V_{2}+(I_{2}-I_{b})\times V_{1}}{I_{2}-I_{1}},$$

where the *V_a_* and *I_b_* refer to the equally spaced voltages and currents, respectively, while *I_a_* and *V_b_* are the corresponding interpolated current and voltage values for the resampled *V_a_* and *I_b_*. Additionally, *I*_1_, *I*_2_, *V*_1_, and *V*_2_ represent the current and voltage values of the nearest data points on the left and right sides, respectively, relative to the resampled points.

The I-V characteristic curves of PV arrays are significantly influenced by environmental factors, so temperature and irradiance need to be included in the data samples to eliminate their effects on the dataset. In addition, dust accumulation on PV panels leads to a reduction in the incident irradiance. Based on the characteristics of PV panels, the amount of irradiance received by the panels is directly related to the short-circuit current [33]. Hence, we propose the short-circuit scale coefficient ($S_{sc}$), with its calculation formula as follows:

(4) $$S_{sc}=\frac{I_{SC}}{[I_{SC\_stc}+\alpha_{rel}(T-T_{stc})]\cdot G/G_{stc}},$$

where *G* denotes the measured irradiance, $G_{stc\;}$represents the irradiance under STC, $I_{SC\_stc}\;$represents the short-circuit current under STC, $T_{stc\;}$represents the temperature under STC, and *α_rel_* is the temperature coefficient of *I_SC_*.

When the feature data are fed directly into the FDD model, the variation in the range of sample features can be too large, resulting in a drop in overall accuracy. Therefore, it is first necessary to normalize the PV array fault samples. In this study, the min–max normalization method is adopted to ensure consistency across the feature range.

### 3.2. The Proposed ResGRU Fault Diagnosis Model

Currently, numerous machine learning techniques are extensively employed for PV fault diagnosis, including the light gradient boosting method (LGBM) [34] and artificial neural network (ANN) [35], among others. Nonetheless, these conventional methods are generally restricted to diagnosing individual faults due to their inability to handle complex compound faults. This limitation prompts the adoption of deep learning methods, which provide enhanced accuracy and generalization capabilities.

**Figure 9:**
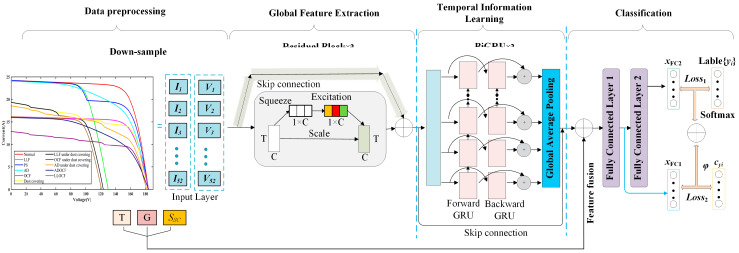
The proposed ResGRU based FDD model structure.

The suggested FDD model for the PV array, seen in Figure 9, comprises three primary blocks: (1) global feature extraction, (2) temporal information learning (TIL), and (3) classification. Initially, the ResNet with SE architecture is exploited to extract the global features from the I-V curves. Next, the TIL block is utilized to mine temporal dynamic features, making it easier to discern the correlations and dependencies between I-V curves. Finally, the classification process is carried out by the fully connected layer. To mitigate the problem of small inter-class distances, we incorporate center loss as a new loss function. In the subsequent sections, we will present a comprehensive explanation of each block.

#### 3.2.1. ResNet with SE Block for Global Feature Extraction

**Figure 10:**
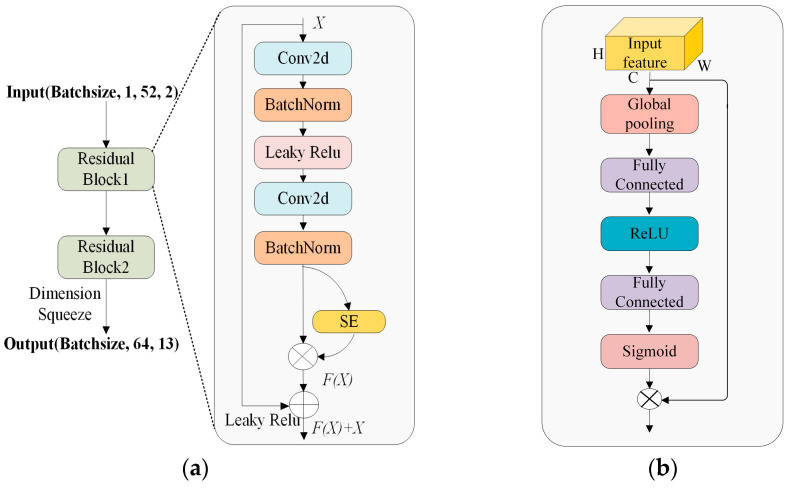
(**a**) Architecture of the ResNet block with SE for global feature extraction; (**b**) architecture of SE block.

In the global feature extraction phase, we employ stacked residual convolutional blocks integrated with SE blocks to capture global features, as shown in Figure 10a. Consequently, the original I-V curve signals are compressed into significantly shorter sequences of local feature vectors. This phase’s input is a two-dimensional matrix composed of the down-sampled I-V curve, and it has a fixed size. The residual blocks are employed for initial fault feature extraction, followed by the addition of SE blocks to enhance useful features and suppress irrelevant ones.

(1) Residual blocks: It is well known that as the depth of a network exceeds a certain threshold, the accuracy no longer increases and may even decrease, making deep neural networks inferior to shallow ones. To address this limitation, the proposed model employs residual blocks to mitigate the gradient vanishing issue in the network [36]. Residual learning accelerates the training process and yields better results than traditional deep neural networks (DNNs). In a residual block, a shortcut connection exists from the input to the output, effectively bypassing one or more convolutional layers. The core operation in this paper is the two-dimensional convolution, which is mathematically expressed as follows:

(5) $$Conv2d(X)=\sum_{p=0}^{P-1}\sum_{q=0}^{Q-1}X_{\left(i+p,j+q\right)}\cdot W_{\left(p,q\right)},$$

where *X* is the input feature map, *i* and *j* denote the spatial row and column indices of the output, *p* and *q* denote the relative positions within the convolution kernel, *W*_(*p*,*q*)_ corresponds to the weight of the convolution kernel at position (*p*,*q*), and *Q* and *P* denote the width and height of the kernel, respectively. Based on this operation, the residual block integrates additional components to further enhance performance. As illustrated in Figure 10a, it consists of two-dimensional convolution (Conv2D) layers, leaky rectified linear units (leaky ReLU) as activation functions, batch normalization (BN) layers, and the SE block. The output of a residual block can be formulated as follows:

(6) $$H(x)=F(x)+W_{x}x,$$

where *H*(*x*) and *x* represent the output and input, *W_x_* is the dimension-matching factor, and the function *F* denotes the mapping relationship from inputs to outputs. The suggested model comprises a total of two residual blocks.

(2) Squeeze and Excitation Block: The SE block essentially recalibrates features along the channel dimension by applying an attention mechanism to different channels [37]. Figure 10b illustrates the configuration of the SE block. By assigning weights to each feature dimension, it focuses more on useful features in the target region and weakens irrelevant features, resulting in more accurate feature information. The SE block consists of three parts: Squeeze, Excitation, and Reweight. We presume that the SE block receives an input *I* ∈ $R^{H\times W\times C}$. Then, we apply global pooling to diminish the feature dimensions, converting input *I* into *I*’ ∈ $R^{1\times 1\times C}$. This process is called “Squeeze”. After that, the channel selection mechanism is parameterized using one ReLU layer and two fully connected layers to amplify useful characteristics while suppressing less relevant ones. This process is called “Excitation”. Finally, we multiply the obtained feature weights with the original features to produce the refined output, which is called “Reweight”. The mathematical expression for the operation of the SE block is given by

(7) $$I^{\prime }=AvgPooling(I)\in R^{1\times 1\times C},$$

where *AvgPooling* (·) is the average global pooling. The obtained features are subsequently reweighted.

(8) $$T=\sigma (W_{2}(\tau (W_{1}(I^{\prime }))))\in R^{1\times 1\times C}$$

where σ(·) and τ(·) represent the sigmoid and relu activation functions, respectively, *W*_1_(·) represents the weight matrices of the first FC layer, and *W*_2_(·) represents the weight matrices of the second FC layer. Finally, the optimized feature weights are multiplied with the original features by matrix multiplication,

(9) $$\overset{\wedge }{I}=T_{1,1,g}\cdot I_{i,j,g},$$

where *i* = 1, 2, …, *H*; *j* = 1, 2, …, *W*; and *g* = 1, 2, …, *C*. The proposed model contains two SE blocks in total.

#### 3.2.2. BiGRU for Temporal Information Learning (TIL)

**Figure 11:**
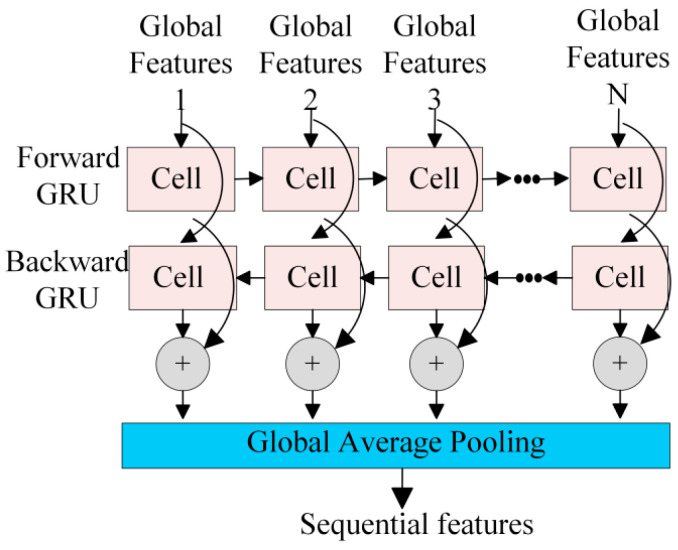
The architecture of BiGRU block. GRU: gated recirculating unit. BiGRU: bidirectional GRU.

After extracting and learning global features, the feature vectors are input into the BiGRU layer, as shown in Figure 11. BiGRU, a variant of the recurrent neural network (RNN), has a strong capacity to learn temporal dependencies and integrates the memory capabilities of long short-term memory (LSTM) networks. Since RNN operates sequentially, we employ a residual structure to speed up the model’s learning process and avoid gradient decay [38]. This approach allows our model to incorporate temporal information from previous input sequences into the features derived from the ResNet.

The TIL module is composed of two BiGRU layers. Each BiGRU layer is followed by batch normalization, which facilitates independent parameter tuning and accelerates model convergence. To further enhance performance, the leaky ReLU activation function is applied after the batch normalization of the BiGRU module. This approach not only overcomes the limitations of relying solely on historical data but also integrates insights into potential future conditions, proving particularly useful. Let *Z* represent the input to the TIL module, with the output *Y*_2_ expressed as follows:

(10) $$Y_{1}=f(BN(GRU(Z)),$$

(11) $$Y_{2}=f(BN(GRU(Y_{1}))+G(Z)),$$

where *GRU*(·) is the GRU network, *BN*(·) is the batch normalization function, *f*(·) is the leaky ReLU activation function, *Y*_1_ is the output after the first GRU module, and *G*(·) is the dimension matching function.

#### 3.2.3. Classification Loss Function and Optimization

Once the network architecture is established, it must be trained using labeled data to learn its internal weights and biases. For classification tasks, the majority of people employ the cross-entropy loss function. However, using cross-entropy loss may lead to the problem of extracting features with small inter-class margins, thereby reducing feature discriminability and compromising classification performance.

The classifier utilizes a combination of center loss and cross-entropy loss as the loss function to optimize the weights. It consists of two fully connected layers, with a BN and ReLU activation function applied after the first layer. Following the first fully connected layer, center loss is also incorporated, and the softmax function is applied after the second fully connected layer. The softmax function, which maps the network’s output features into the interval (0, 1), is defined as follows:

(12) $$P_{i}=\frac{e^{x_{i}}}{\sum_{k=1}^{K}e^{x_{k}}},i=1,2,\ldots K,$$

where *x_i_* represents the unnormalized output of the *i*th class produced by the network, *K* denotes the total number of categories, and $P_{i}$ is the prediction probability by the softmax function. Then, the cross-entropy loss can be formulated as follows:

(13) $$Loss_{1}=-\sum_{i=1}^{N}y_{i}\log P_{i},$$

where N denotes the mini-batch size and $y_{i}$ denotes the true label of the sample.

Next, we introduce center loss [39], which is derived by measuring the distance between features of the same class and their corresponding centers. Its formulation can be expressed as follows:

(14) $$Loss_{2}=\frac{1}{2}\sum_{i=1}^{N}{\left\|x_{i}-c_{y_{i}}\right\|}_{2}^{2},$$

where $c_{y_{i}}$ is the center of the sample of category${\;y}_{i}$. Furthermore, to prevent overfitting, we incorporated a regularization term into the original loss function.

Finally, the loss function of the proposed model is expressed as follows:

(15) $$Loss=Loss_{1}+\varphi Loss_{2}+\lambda \sum_{W}W^{2},$$

where φ denotes the hyperparameter of the center loss function, with its values constrained within the range of [0, 1]. This parameter is utilized to control the contribution of the center loss function to the overall loss. λ represents the regularization coefficient.

To address the issue of slow convergence caused by the fixed learning rate in the Adam optimizer, piecewise linear learning is introduced. This approach enhances network convergence by initially boosting the learning rate during early training and subsequently reducing it in later stages, guiding the network more efficiently to the optimal solution. Table 4 presents the specific hyperparameters of the proposed model. Table 5 presents the detailed structure of the proposed model, where the input is a 52 × 2 data matrix derived from original I-V curve data. It can be seen that *G*, *T*, and *S_sc_* are fused with the previous features in the feature fusion layer to reduce computation.

### 3.3. The General Process for Fault Diagnosis

**Figure 12:**
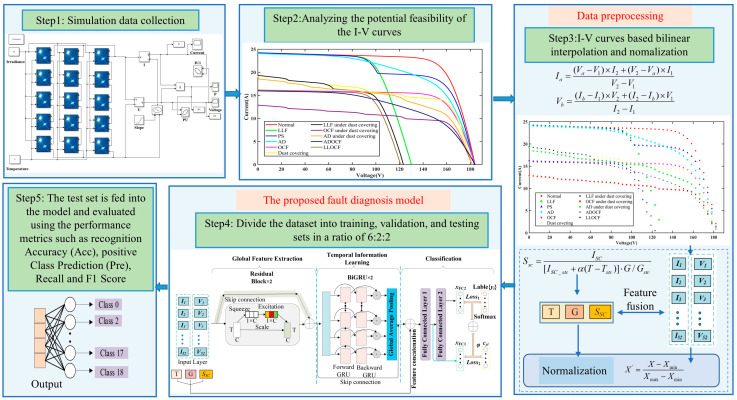
The specific process framework for fault diagnosis.

In order to accurately diagnose PV faults, this paper presents an intelligent diagnostic framework utilizing the ResGRU model. Figure 12 illustrates the proposed diagnostic framework and elaborates on the application process of this method. The process involves five key steps, which are outlined as follows:

Step 1: The Simulink simulation platform is used to model a small-scale PV power generation system. Within this platform, various fault modes are configured to obtain I-V curves corresponding to various fault conditions.

Step 2: By analyzing the I-V curves under various fault conditions, it is evident that the curves vary between faults, indicating the potential viability of using I-V curves for FDD.

Step 3: The bilinear interpolation sampling method is employed on the I-V curves to minimize data redundancy and enhance model training speed. Considering the significant influence of environmental factors on I-V curves, the *T*, *G*, and $S_{sc}$ are fused with the previous features in the feature fusion layer. To further eliminate numerical differences among different electrical parameters, the input data are normalized.

Step 4: The dataset is split into training, validation, and testing subsets. The training subset is utilized to train the ResGRU model, while the validation subset is employed for model optimization.

Step 5: The testing set derived from Step 4 was input into the model, and its performance was then evaluated.

## 4. Results and Discussion

In an actual environment, obtaining large-scale fault data samples for PV arrays is challenging due to the uncontrollable nature of temperature and irradiance. Furthermore, conducting fault experiments on actual PV arrays could result in damage, thus carrying inherent risks. To ensure the reliability of data samples, current research primarily relies on simulation experiments to acquire data samples. Given the irreparable damage caused by faulty experiments, noise and measurement errors were added to simulate real-world conditions, thereby generating both noiseless and noisy data samples for testing the proposed model’s effectiveness.

The fault settings align with the descriptions provided in Section 2.3. These faults selected are primarily based on their substantial effect on PV power generation output and their frequent occurrence [40]. Table 6 presents the fault types analyzed in this study. The faults are categorized based on the dust coverage level of the PV panels into three groups: clean, DC1, and DC2. In the clean state, four common individual faults are investigated: OCF, LLF, PS, and AD, along with two compound faults: ADOCF and LLOCF. LLF is further divided into two categories based on fault severity: one module short-circuited (LL1) and two module short-circuited (LL2). Similarly, PS is subdivided into two categories based on shading severity: one module shaded (PS1) and two module shaded (PS2). In the DC1 state, the faults include OCF, LL1, LL2, and AD, all occurring under the DC1 condition. The fault types in the DC2 state are identical to those in the DC1 state, with the only difference being the dust coverage level of the PV panels. The proposed ResGRU model is developed using the pytorch framework. The server utilized for implementation is equipped with an Intel i5-12400F CPU, an NVIDIA RTX 3060 GPU, and 16 GB of RAM.

### 4.1. Data Acquisition and Description

In this paper, the simulation model presented in Figure 3 is employed to simulate various faults. The control voltage source utilizes a ramp signal to continuously monitor the output voltage of the PV array. The parameters for simulating different faults are configured as follows: LL1 and LL2 are simulated by shorting one or two modules, respectively. For PS, PS1, and PS2 are simulated by setting the irradiance gain amplifier of one or two modules to a value of 0.5, respectively. Additionally, OCF is simulated by disconnecting one string from the array. And AD is simulated by adding a 10 Ω resistor in series with one of the strings in the PV array. Dust accumulation is treated as a specific type of shadowing that affects all PV modules [41]. Due to non-uniform dust coverage among modules, DC1 and DC2 are simulated by randomly setting the irradiance gain amplifier of all modules in the PV array to [0.8, 1] and [0.6, 0.8], respectively. Finally, the parameters of the two relevant individual faults are combined to simulate the corresponding compound faults.

To enhance the model’s applicability across various environmental circumstances, different temperatures and irradiance levels are assigned to each fault. The temperature range is established from 10 °C to 70 °C with a step size of 3 °C, while irradiance values range from 200 W/m^2^ to 1000 W/m^2^ with a step size of 20 W/m^2^. Consequently, there are 861 simulation samples for each operational condition, resulting in a total of 16,359 data samples. The data samples of each type are randomly divided into training, validation, and testing subsets in a ratio of 6:2:2, with the corresponding sample sizes for each subset summarized in Table 6.

### 4.2. Evaluation Metrics

In this paper, we employ widely adopted metrics such as recall, precision, accuracy, and F1-score as standards to assess the effectiveness of the proposed method [42,43]. These metrics’ calculation formulas are as follows:

(16) $$R=\frac{T_{P}}{T_{P}+F_{N}},$$

(17) $$P=\frac{T_{P}}{T_{P}+F_{P}},$$

(18) $$A=\frac{T_{P}+T_{N}}{T_{P}+F_{P}+F_{N}+T_{N}},$$

(19) $$F=\frac{2\cdot (P\cdot R)}{P+R},$$

where *R*, *P*, *A,* and *F* represent recall, precision, accuracy, and F1-score, respectively. *T_P_* denotes the true positives, *F_N_* denotes the false negatives, *T_N_* denotes the true negatives, and *F_P_* denotes the false positives.

To enhance interpretability in multiclass classification, we use the macro-average method for calculating *R*, *P*, and *A*. This approach computes the unweighted mean across all classes, ensuring equal treatment of each class and penalizing poor performance in minority classes.

### 4.3. Case 1: Performance Evaluation on Noiseless Dataset

#### 4.3.1. Feature Visualization

**Figure 13:**
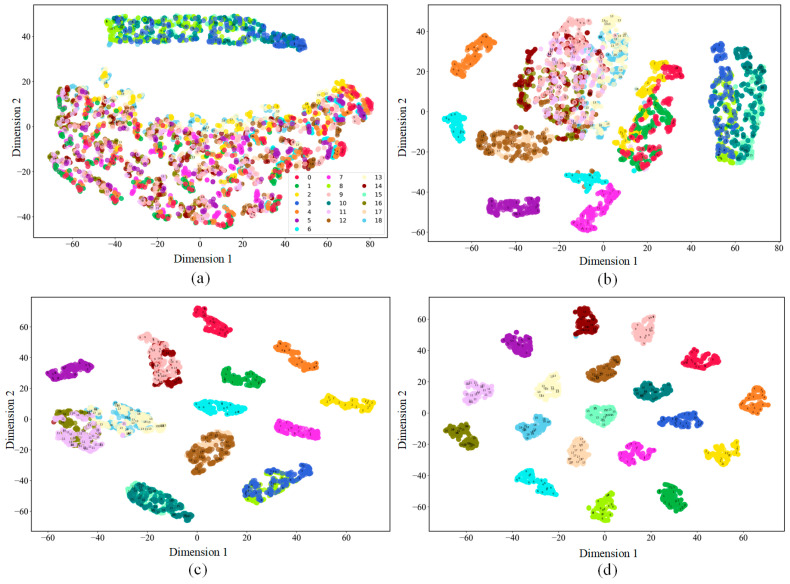
Visualization results of the noiseless dataset: (**a**) raw data; (**b**) data processed with ResNet and SE; (**c**) data processed with BiGRU; (**d**) output data.

To validate the efficacy of the proposed feature extraction method and model parameter construction, we employ t-distributed stochastic neighbor embedding (t-SNE) to graphically depict the sample features extracted by the model [44], which have been reduced to two dimensions for visualization. The visualization results of sample features are depicted in Figure 13.

Figure 13a illustrates the visualization of the raw input data, where categories are scattered with low clustering, making it hard to distinguish them. Figure 13b shows the visualization of the data processed by ResNet with the SE block, where a few clusters form, but significant overlap remains, indicating the necessity for better feature extraction. Figure 13c displays the visualization results after dynamic feature extraction with BiGRU, where more categories form distinct clusters, though some overlap remains. Figure 13d presents the final output data visualization after FC_2, with categories clearly separated and most data points accurately classified. This stepwise feature extraction visualization shows that the proposed method enhances classification performance by improving feature recognition capability. The FDD model exhibits excellent performance on PV array fault data.

#### 4.3.2. Analyses of Training and Testing Results

**Figure 14:**
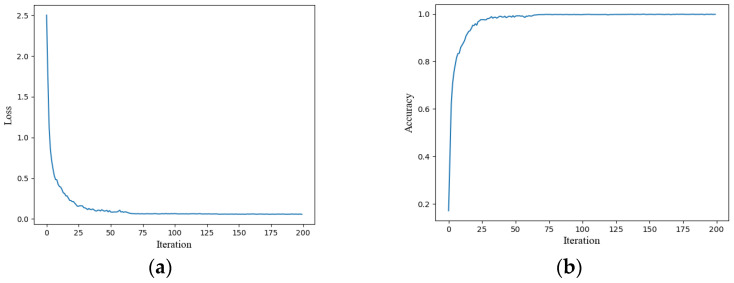
Training process of the ResGRU model. (**a**) Training loss curve; (**b**) training accuracy curve.

**Figure 15:**
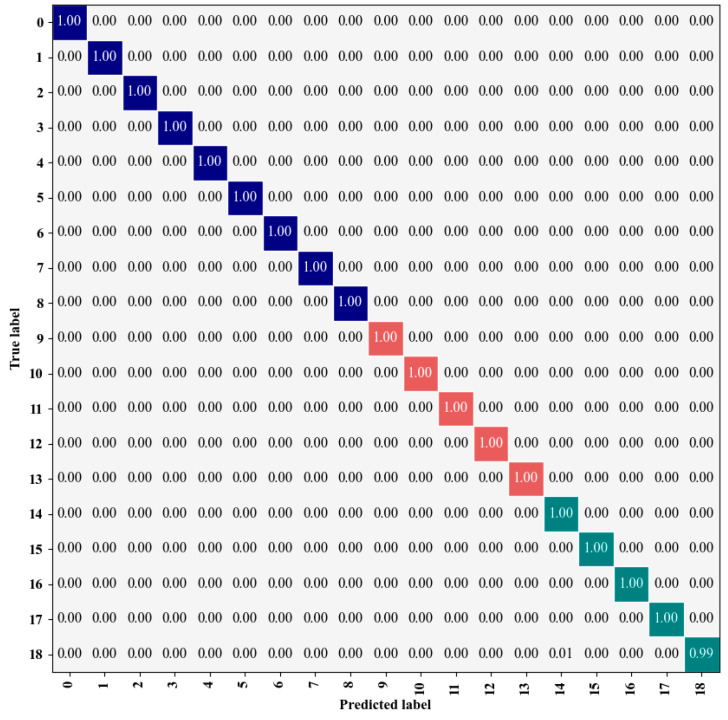
Standardized confusion matrix on noiseless dataset. The matrix uses three distinct colors to represent the classification results under clean (blue), DC1 (red), and DC2 (green) conditions.

Figure 14a,b show the classification loss curve and accuracy curve, respectively, following 200 iterations. The final model achieved a training accuracy of 99.9%. Initially, when the iteration count is below 90, both accuracy and loss change rapidly, with quick convergence. After the iteration count surpasses 90, both loss and accuracy curves exhibit a tendency to stabilize. The trained model is then deployed for the purpose of predicting the test set categories, with a standardized confusion matrix employed to evaluate the classification performance. Figure 15 shows the standardized confusion matrix, which is generated by normalizing the matrix row-wise. The matrix values represent the likelihood of assigning samples to specific labels, while the diagonal values represent the proportion of correctly classified instances for each category, effectively reflecting the recall rate. Furthermore, the confusion matrix employs three distinct colors to represent the classification results under clean, DC1, and DC2 conditions. It is evident that, except for a slight misjudgment in the last category, the model achieves a recall rate of 1 for all other categories. In summary, the proposed method demonstrates a high level of recognition accuracy.

### 4.4. Case 2: Performance Evaluation on Noisy Dataset

Due to the limitations in the accuracy and capability of equipment, the data collected often contain noise. In contrast, the generation of simulation curves is conducted under conditions as close to ideal as possible, without any measurement errors or variations. To closely approximate real-world scenarios and train classifiers that reflect actual measurements, environmental noise and measurement errors are incorporated into the previously noiseless data. The noise is modeled using a normal distribution, with a signal-to-noise ratio (SNR) set at 35 dB, and random errors are set with variances of 0.5% for both V and I. These values were derived by combining the uncertainties specified in the literature and technical reports [45,46], to simulate real-world conditions. The method for generating noisy data in this study is described as follows:

(20) $$Noisy\;signal=Noiseless\;signal+random(\sigma^{2},(m,n)),$$

where *random* () refers to a predefined Python function that generates a matrix of white Gaussian noise with a specified number of *m* × *n.* The variance $\delta^{2}\;$is determined by the signal power, and the desired signal-to-noise ratio (SNR) is determined as follows:

(21) $$\sigma^{2}=\frac{P_{signal}}{10^{\frac{SNR}{10}}}.$$

#### 4.4.1. Analyses of Training and Testing Results

**Figure 16:**
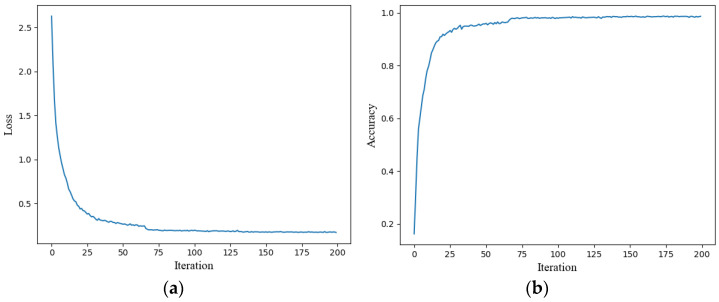
Training results of 200 iterations on noisy dataset. (**a**) Loss curve; (**b**) accuracy curve.

**Figure 17:**
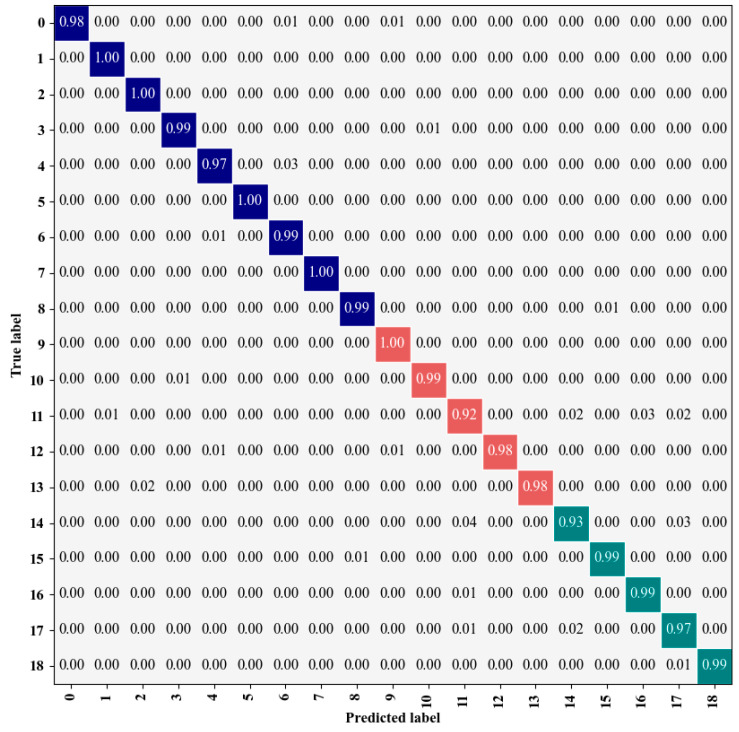
Standardized confusion matrix on noisy dataset. The matrix uses three distinct colors to represent the classification results under clean (blue), DC1 (red), and DC2 (green) conditions.

Figure 16a,b show the model’s loss and accuracy curves after 200 iterations on the training set. The final model achieved a training accuracy of 98.60%. It is evident that when the iteration count is below 100, the loss and accuracy of the training set converge rapidly. Once the iterations exceed 100, both loss and accuracy become more stable. Figure 17 presents the standardized confusion matrix, which also employs three distinct colors to represent the classification results under clean, DC1, and DC2 conditions, demonstrating that the model achieves optimal fault classification performance under clean conditions, with recall rates ranging from 0.97 to 1.0. Specifically, categories 1, 2, 5, and 7 achieved 100% classification accuracy. However, under DC1 and DC2 conditions, fault classification accuracy decreases compared to the clean condition. This may be attributed to noise, which complicates the compound fault features. Nevertheless, the fault identification accuracy remains high in most cases.

To further demonstrate the model’s performance across various fault types, Table 7 presents detailed metrics (recall, precision, and F1-score) for each fault under different levels of dust coverage (Clean, DC1, and DC2). Notably, the model achieved approximately 98.21% accuracy on the noisy dataset, demonstrating the robustness of the proposed method in noisy environments and its effectiveness in improving PV array fault diagnosis.

#### 4.4.2. Ablation Study

The proposed model comprises ResNet, SE, and BiGRU modules, in addition to a center loss function. We performed an ablation research on the noisy dataset to evaluate the contribution of each module to the model’s performance, as illustrated in Table 8. We specifically developed four model variants, with the first three variants not using the center loss function. The models are as follows:

- M1: ResNet module only.
- M2: ResNet and BiGRU module without SE.
- M3: Training ResNet, BiGRU, and SE together, without the center loss function.
- M4: Training ResNet, BiGRU, and SE together with the center loss function.

The results of the ablation study presented in Table 8 explicitly show that the proposed model (M4) achieves optimal performance on the noisy dataset. Firstly, the M1 framework achieves a macro-average accuracy of 96.50%. This indicates that while ResNet alone provides a certain level of classification capability, it is limited in its ability to capture complex data features, necessitating further enhancements. Secondly, the incorporation of the BiGRU module in the M2 model results in substantial enhancements in performance metrics. This suggests that the BiGRU module can capture and retain temporal dynamics effectively, enabling the model to discern the interrelations and dependencies among I-V curves. Thirdly, the M3 model further integrates the SE module, yielding further improvements in performance metrics. This indicates that the SE module enhances the model’s capacity to prioritize critical features while reducing the influence of non-essential features, thereby improving overall performance. Finally, the M4 model combines the strengths of ResNet, BiGRU, and SE modules, while also incorporating a center loss function. By constraining intra-class distances, the center loss function significantly improves intra-class compactness, further enhancing the model’s discriminative ability and achieving optimal overall performance.

**Figure 18:**
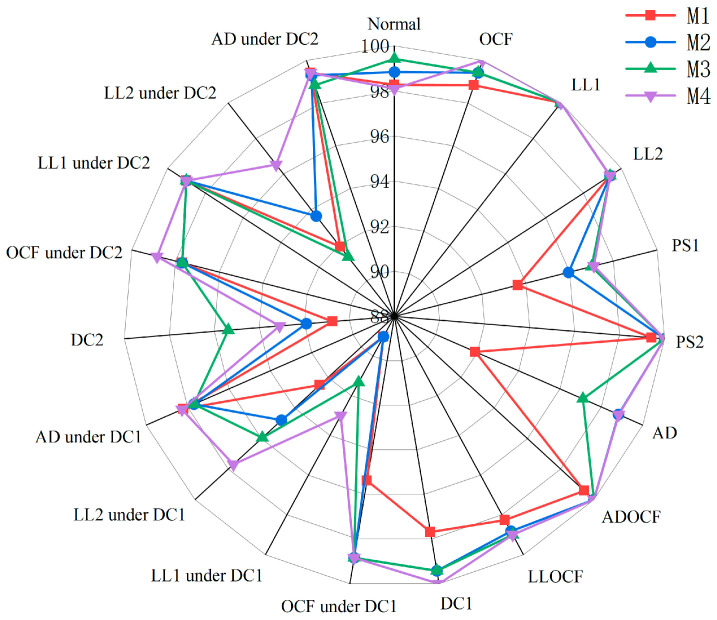
Radar chart illustrating the accuracy of four model variants across different fault types.

To provide a more intuitive comparison of the models’ specific performance under different fault types, we constructed a radar plot that contrasts the accuracy of each model for each fault scenario. Figure 18 shows that the M4 model outperforms other models in most categories. Specifically, the M4 model achieved 100% accuracy for fault types such as OCF, LL1, ADOCF, PS2, and DC1, significantly outperforming other model variants. This underscores the superior ability of the M4 model to capture complex fault features and its resilience across various conditions, making it highly effective for practical fault diagnosis.

#### 4.4.3. Evaluation of Loss Function

This section evaluates the influence of the center loss coefficient φ on the performance of the proposed model. Table 9 demonstrates the impact of varying φ values on the model’s performance. Without center loss (φ = 0), the model achieves an accuracy of 97.87%. As φ increases, the performance improves, demonstrating the effectiveness of center loss in enhancing inter-class separability and intra-class compactness. However, when φ is set too high, performance begins to decline. This decline may be due to the model overly emphasizing intra-class variation, which can result in the centers of different classes being drawn closer together, thereby reducing inter-class separability. Consequently, selecting an optimal φ value is crucial for maximizing the accuracy of fault diagnosis.

#### 4.4.4. Comparison with Other Methods

To assess the effectiveness of the proposed method, we conduct both qualitative and quantitative comparisons with the other four methods from references [2,27,47,48]. Table 10 provides a summary of the qualitative analysis, while Table 11 shows the results of the quantitative analysis. This comparison aims to illustrate the benefits of the proposed method regarding diagnostic metrics while also emphasizing its overall superior performance through an analysis of the similarities and differences among various methods. It is important to note that we utilized the relevant methodologies from references [2,27,47,48] on the identical dataset employed in this investigation, rather than relying on the accuracy metrics documented in those papers. That is to say, to ensure a fair comparison, the training, validation, and test sets in Table 11 are consistent.

As shown in Table 10, Laurino et al. [2] proposed a method using an artificial neural network (ANN) to classify sixteen operating states of PV panels under various conditions, including open-circuit fault, short-circuit fault, and normal state, etc. However, this method does not take dust coverage into account. Chen et al. [27] combined the ResNet network with I-V curves for PV fault diagnosis. This method identifies individual faults such as short-circuit fault, open-circuit fault, partial shading, and abnormal aging but does not address complex compound faults or dust coverage. Lin et al. [47] proposed a multi-scale SE-ResNet network, using environmental data and raw I-V curves to detect faults, including partial shading and compound faults under varying levels of dust accumulation. However, it does not consider compound faults in clean environments. Amiri et al. [48] used a random forest classifier for fault detection but did not consider the impact of compound faults and dust coverage. In addition, the method in reference [48] relies on manually extracting features from the I-V curves, heavily depending on expert knowledge, and lacks automation capabilities.

To guarantee the experiments’ fairness, the hyperparameters of other models are set to be consistent with those in this study. Since Amiri et al. [48] identified fewer faults and the features used were inadequate for handling both single and compound fault recognition, we directly use the I-V curves and environmental factors as inputs for a fair comparison. Additionally, we perform comparative analysis on alternative models such as ResNet-BiLSTM, ResNet-LSTM, and ResNet-GRU. These models share the same architecture as our proposed ResGRU model, with the sole difference being the substitution of the BiGRU module with BiLSTM, LSTM, and GRU, respectively. The experimental results of these models are shown in Table 11. The results show that the proposed method outperforms all other approaches, achieving over 98% in all evaluated metrics and significantly exceeding the performance of alternative methods.

**Figure 19:**
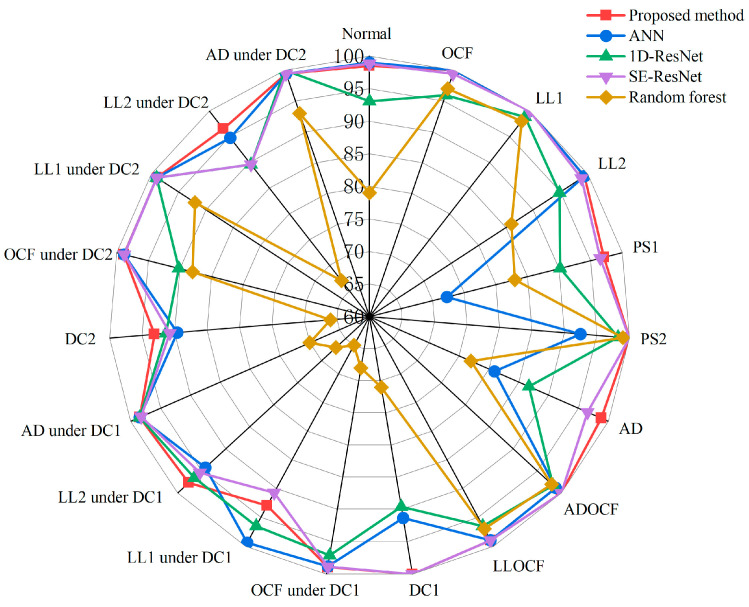
Radar chart illustrating the accuracy of five FDD models across different fault types.

Moreover, to more intuitively demonstrate the performance of each model under different fault types, we plot the radar chart shown in Figure 19. This chart illustrates the classification accuracy of the proposed model compared to [2,27,47,48] across different fault types. Specifically, the proposed model performs exceptionally well in most fault types, achieving 100% classification accuracy under typical fault conditions such as OCF, LL1, ADOCF, and PS2. Additionally, the proposed model remains stable and significantly outperforms other methods under complex fault conditions such as LL2 under DC2 and LL2 under DC1. In contrast, the Random forest model performs the worst across all fault types, with an average accuracy of only 82%, and only the PS2 fault is accurately identified. Overall, the proposed model outperforms existing methods and shows better classification performance under most fault conditions.

## 5. Conclusions

This paper proposes a fault diagnosis approach for PV arrays, which integrates ResNet and BiGRU networks. The method not only addresses single faults and partial shading conditions but also comprehensively evaluates compound faults under varying degrees of dust coverage. Initially, ResNet is employed to extract both global and detailed features from the sequences, followed by BiGRU to retain and process the temporal dynamic features, thereby enabling precise fault classification. To mitigate data redundancy, the raw I-V curve data are down-sampled before being fed into the network. Subsequently, the current and voltage data are concatenated into a two-dimensional matrix to serve as input for the feature extraction network, while temperature, irradiance, and the short-circuit scale coefficient are directly input into the feature fusion layer to reduce computation. Furthermore, SE modules are integrated into the ResNet network to enhance relevant features while suppressing irrelevant ones. Finally, a center loss function is introduced in the classification module to further enhance feature discriminability and improve classification performance. The suggested method has higher fault classification ability, as validated by numerical simulations and noisy experiments, obtaining accuracy rates of 99.94% and 98.21%, respectively. The suggested fault diagnosis model has superior diagnostic performance relative to alternative techniques. This research offers substantial direction for future investigations focused on improving the dependability and fault diagnostics of PV systems.

Despite the high fault identification capability of the proposed method, forthcoming studies should concentrate on the subsequent two domains: (1) Current studies on PV faults primarily focus on PV arrays, with limited research on other system components, such as converters and batteries. (2) Experiments need to be conducted on actual PV systems to collect data under various fault conditions and further validate the proposed methods. (3) A framework for intelligent remote monitoring of fault diagnosis in PV systems is planned for future development.

## Figures and Tables

**Table 1:** Comparison of the previous methods for PV fault diagnosis.

Ref.	Methods	Advantages	Disadvantages
[9,10,11,12,13]	Model-driven approaches	It has relatively low hardware requirements and offers a certain degree of interpretability.	When multiple faults occur, the model becomes more complex, requiring advanced diagnostic algorithms to extract deep feature insights.
[16,17,18]	Image-based methods	It is effective for the prompt detection and localization of faults or defects in PV systems.	These images often require collection via high-resolution cameras and UAVs, resulting in considerable expenses.
[19,20,21]	String current or voltage-based methods	The method enables real-time monitoring of string current and voltage, allowing for continuous data collection.	Most sensors can result in higher maintenance costs. Moreover, different types of faults may lead to similar changes in current and voltage.
[22,23,24,25]	I-V curves (using extracted key features)	The fault diagnosis model is relatively simple, requiring only partial I-V curve data, thus optimizing computational resources.	These methods rely heavily on manual feature extraction and expert knowledge, which lack automation and may overlook valuable features.
[26,27]	I-V curves (using the entire curves)	The method eliminates the need for manual feature extraction, providing comprehensive fault information.	It tends to need more complex deep learning models to accurately identify multiple faults.

**Table 2:** Specification sheet of ASMS-220P PV module.

Parameters	Value	Parameters	Value
*I_SC_*	8.08 A	*V_MPP_*	30 V
*V_OC_*	36.8 V	*α* *_rel_*	0.038%/C
*I_MPP_*	7.35 A	*β* *_rel_*	−0.336%/C

**Table 3:** Specification sheet of PV array.

Parameters	Value	Parameters	Value
*I_SC_*	24.24 A	*V_MPP_*	150 V
*V_OC_*	184 V	*I_MPP_*	22.05 A

**Table 4:** Hyperparameter settings for training the proposed ResGRU model.

Hyperparameters	Value
lr	0.001
$\beta_{1}$	0.9
$\beta_{2}$	0.999
Epoch	200
Batch size	128
Weight decay rate λ	0.001
Center loss φ	0.005

**Table 5:** Detailed structure of the proposed ResGRU model.

Layer/Block	Output Shape	Number of Filters	Kernel Size	Stride	Activation Function
Dimension Unsqueeze	1×52×2	-	-	-	-
Residual Block1 Conv2d_1:	32×52×2	32	3×1	1	Leaky Relu
Residual Block1 Conv2d_2:	32×26×2	32	3×1	2	Leaky Relu
Residual Block2 Conv2d_3:	32×26×2	32	3×1	1	Leaky Relu
Residual Block2 Conv2d_4:	32×13×2	32	3×1	2	Leaky Relu
Dimension Squeeze	64×13	-	-	-	-
BiGRU1	128×13	64	-	-	Leaky Relu
BiGRU2	128×13	64	-	-	Leaky Relu
AdaptiveAvgPool1d	128×1	-	-	-	-
Feature Fusion	131×1	-	-	-	-
Fully Connected1 (FC_1)	64	-	-	-	ELU
Fully Connected2 (FC_2)	19	-	-	-	Softmax

**Table 6:** Overview of simulated data sample distribution.

Operating Condition	Fault Type	Class Label	Sample		
			Training Set	Validating Set	Test Set
Clean	Normal	0	517	173	171
	OCF	1	517	173	171
	LL1	2	517	173	171
	LL2	3	517	173	171
	PS1	4	517	173	171
	PS2	5	517	173	171
	AD	6	517	173	171
	ADOCF	7	517	173	171
	LLOCF	8	517	173	171
DC1	DC1	9	517	173	171
	OCF under DC1	10	517	173	171
	LL1 under DC1	11	517	173	171
	LL2 under DC1	12	517	173	171
	AD under DC1	13	517	173	171
DC2	DC2	14	517	173	171
	OCF under DC2	15	517	173	171
	LL1 under DC2	16	517	173	171
	LL2 under DC2	17	517	173	171
	AD under DC2	18	517	173	171

**Table 7:** Classification report of the fault diagnosis model.

Operating Condition	Fault Type	Class Label	Metrics		
			Precision	Recall	F1-Score
Clean	Normal	0	1.0000	0.9799	0.9898
	OCF	1	0.9943	1.0000	0.9971
	LL1	2	0.9830	1.0000	0.9914
	LL2	3	0.9885	0.9942	0.9914
	PS1	4	0.9825	0.9711	0.9767
	PS2	5	1.0000	1.0000	1.0000
	AD	6	0.9607	0.9884	0.9744
	ADOCF	7	1.0000	1.0000	1.0000
	LLOCF	8	0.9884	0.9884	0.9884
DC1	DC1	9	0.9720	1.0000	0.9860
	OCF under DC1	10	0.9942	0.9884	0.9913
	LL1 under DC1	11	0.9302	0.9280	0.9291
	LL2 under DC1	12	1.0000	0.9769	0.9883
	AD under DC1	13	1.0000	0.9827	0.9913
DC2	DC2	14	0.9641	0.9306	0.9471
	OCF under DC2	15	0.9884	0.9884	0.9884
	LL1 under DC2	16	0.9884	0.9884	0.9884
	LL2 under DC2	17	0.9435	0.9653	0.9543
	AD under DC2	18	1.0000	0.9942	0.9971
Macro avg			0.9822	0.9821	0.9820
Accuracy (%)			98.21		

**Table 8:** Ablation study conducted on noisy dataset.

Model	Accuracy (%)	Precision (%)	Recall (%)	F1-Score (%)
M1	96.50	96.50	96.50	96.49
M2	97.59	97.61	97.59	97.59
M3	97.87	97.89	97.87	97.87
M4	98.21	98.22	98.21	98.20

**Table 9:** Classification accuracies with different center loss parameters.

φ	0	0.0001	0.0005	0.001	0.005	0.01	0.05	0.1
Accuracy (%)	97.87	98.02	97.87	98.05	98.21	97.87	97.78	97.29

**Table 10:** Comparison of the proposed algorithm with other methods in [2,27,47,48].

Case	Proposed Method	[2]	[27]	[47]	[48]
Year	2024	2022	2019	2022	2024
Input data	$\mathrm{I-V}\;\mathrm{curve},\;T\;\mathrm{and}\;G,\;S_{sc}$	I-V curve	I-V curve, *T*, and *G*	$\mathrm{I-V}\;\mathrm{curve},\;T\;\mathrm{and}\;G,\;S_{sc}$	$T,\;G,\;I_{mpp}$ $,\;V_{mpp}$ $,\;\mathrm{and}\;P_{mpp}$
Classification model	ResGRU	ANN	1D-ResNet	SE-ResNet	Random forest
Hybrid faults consideration	√	√	×	√	×
Dust coverage consideration	√	×	×	√	×

**Table 11:** Comparison of various FDD models on noisy datasets.

Model	Accuracy	Precision	Recall	F1-Score
Proposed method	98.21%	98.22%	98.21%	98.20%
[2]	95.44%	95.48%	95.44%	95.40%
[27]	94.46%	94.52%	94.46%	94.47%
[47]	97.41%	97.42%	97.41%	97.41%
[48]	82.00%	83.00%	82.00%	82.00%
ResNet-BiLSTM	98.05%	98.07%	98.05%	98.05%
ResNet-LSTM	97.51%	97.51%	97.51%	97.50%
ResNet-GRU	97.41%	97.45%	97.41%	97.40%

## Data Availability

Data is contained within the article.
